# *BRCA1/2* testing among young women with breast cancer in Massachusetts, 2010–2013: An observational study using state cancer registry and All‐Payer claims data

**DOI:** 10.1002/cam4.4648

**Published:** 2022-03-21

**Authors:** Lydia E. Pace, John Z. Ayanian, Robert E. Wolf, Richard Knowlton, Susan T. Gershman, Summer Sherburne Hawkins, Nancy L. Keating

**Affiliations:** ^1^ Brigham and Women's Hospital Boston Massachusetts USA; ^2^ Harvard Medical School Boston Massachusetts USA; ^3^ Institute for Healthcare Policy and Innovation University of Michigan Ann Arbor Michigan USA; ^4^ Massachusetts Cancer Registry, Office of Data Management and Outcomes Assessment, Office of Population Health, Massachusetts Department of Public Health Boston Massachusetts USA; ^5^ Boston College School of Social Work Newton Massachusetts USA

**Keywords:** *BRCA1/2*, breast cancer, disparities, Massachusetts, Medicaid

## Abstract

**Background:**

Testing for *BRCA1/2* pathogenic variants is recommended for women aged ≤45 years with breast cancer. Some studies have found racial/ethnic and socioeconomic disparities in testing. We linked Massachusetts' All‐Payer Claims Database with Massachusetts Cancer Registry data to assess factors associated with *BRCA1/2* testing among young women with breast cancer in Massachusetts, a state with high levels of access to care and equitable insurance coverage of breast cancer gene (*BRCA)* testing.

**Methods:**

We identified breast cancer diagnoses in the Massachusetts Cancer Registry from 2010 to 2013 and linked registry data with Massachusetts All‐Payer Claims Data from 2010 to 2014 among women aged ≤45 years with private insurance or Medicaid. We used multivariable logistic regression to examine factors associated with *BRCA1/2* testing within 6 months of diagnosis.

**Results:**

The study population included 2424 women; 80.3% were identified as non‐Hispanic White, 6.4% non‐Hispanic Black, and 6.3% Hispanic. Overall, 54.9% received *BRCA1/2* testing within 6 months of breast cancer diagnosis. In adjusted analyses, non‐Hispanic Black women had less than half the odds of testing compared with non‐Hispanic White women (adjusted odds ratio [OR] = 0.45, 95% CI = 0.31, 0.64). Medicaid‐insured women had half the odds of testing compared with privately‐insured women (OR = 0.51, 95% CI = 0.41, 0.63). Living in lower‐income areas was also associated with lower odds of testing. Having an academically‐affiliated oncology clinician was not associated with testing.

**Conclusion:**

Socioeconomic and racial/ethnic disparities exist in *BRCA1/2* testing among women with breast cancer in Massachusetts, despite equitable insurance coverage of testing. Further research should examine whether disparities have persisted with growing testing awareness and availability over time.

## Graphical abstract

This study examines BRCA1/2 testing rates among women aged ≤45 years with breast cancer in Massachusetts in 2010–2013 using linkage between the state's cancer registry and All‐Payer Claims Data. Despite insurance coverage and guidelines recommending testing, just over half of privately‐ or Medicaid‐insured women received testing. Odds of testing were lower among Black women and Medicaid‐insured women compared with White and privately‐insured women, respectively, and lower among women living in lower‐income compared with higher‐income areas.

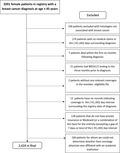

## 1 BACKGROUND

However, disparities in receipt of recommended testing have been identified. In particular, despite there being no evidence to suggest that Black women with breast cancer have lower rates of *BRCA1/2* pathogenic variants than White women,^3^ studies from several states have found that Black women with breast cancer have lower rates of indicated testing than White women.^4^, ^5^, ^6^ These inequities are particularly concerning given that Black women are more likely to be diagnosed with breast cancer before age 40 than White women and have higher breast cancer mortality rates.^7^, ^8^

The contribution of insurance coverage and access to racial/ethnic disparities in *BRCA* testing is not well understood. To examine whether disparities exist in a population with adequate health insurance coverage of genetic testing and access to care, we examined *BRCA* testing among women aged ≤45 years who were newly diagnosed with breast cancer in Massachusetts. Massachusetts has the fewest uninsured residents of any state, the highest ratio of primary care physicians to residents,^9^ and Massachusetts' Medicaid program has consistently covered *BRCA1/2* testing in individuals with cancer as recommended by NCCN guidelines. To examine a diverse and representative population of Massachusetts women with cancer, we employed a distinctive linkage between the state's cancer registry and All‐Payers Claims Data (APCD) to assess *BRCA1/2* testing among all privately‐ and Medicaid‐insured women aged ≤45 diagnosed with breast cancer in Massachusetts between 2010 and 2013.

## 2 METHODS

### 2.1 Data sources

We linked data on breast cancer diagnoses in 2010–2013 from the Massachusetts Cancer Registry (MCR) with the Massachusetts APCD^10^ from 2010 to 2014, which included claims data from all commercial payers and Medicaid in Massachusetts. Linkage was achieved using a probabilistic record linkage program, Link Plus, developed by the Centers for Disease Control.^11^ A detailed description of linkage processes is available in Appendix S1.

### 2.2 Study population

**FIGURE 1:**
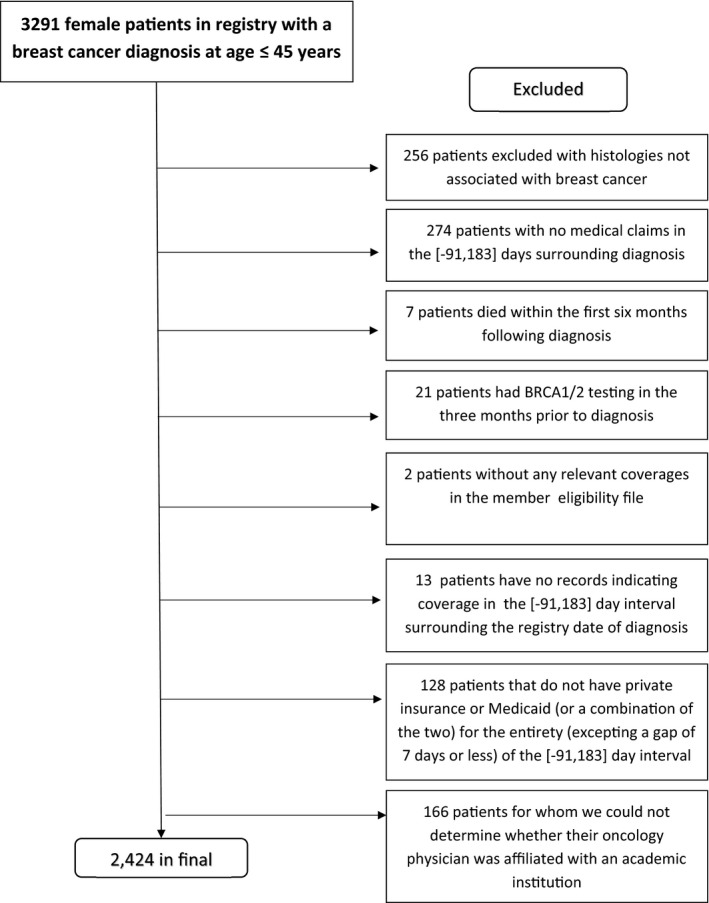
Flow diagram of cohort‐building process

Our study cohort included women in Massachusetts aged 18–45 who were diagnosed with breast cancer during 2010–2013 and were enrolled in commercial insurance or Medicaid at the time of their diagnosis based on APCD enrollment files. Cohort development is shown in Figure 1. Specifically, women were included if they were continuously enrolled in insurance for at least 3 months before their first breast cancer diagnosis and were alive and continuously enrolled through 6 months following diagnosis to ascertain BRCA testing. We excluded women who received *BRCA1/2* testing during the 3 months before diagnosis (*n* = 21). For our primary analysis, we also excluded women for whom we could not identify whether their hospital or physician was academically affiliated (*n* = 166); this typically occurred when we could not reliably identify the physician responsible for their surgical or medical oncology care. Most of these patients had few treatment‐related claims in the APCD.

### 2.3 BRCA1/2 testing

Our primary outcome was the receipt of *BRCA1/2* testing within 6 months following their breast cancer diagnosis based on a claim with Common Procedural Terminology (CPT) code 81,211, 81,212, 81,213, 81,214, 81,215, 81,216, or 81,217 or Healthcare Common Procedure Coding System (HCPCS) code S3818, S3819, S3820, S3821, S3822, or S3823. Although guidelines do not specify recommended timing of BRCA1/2 testing following a breast cancer diagnosis, we chose 6 months as the time interval for this outcome because testing is most helpful if performed before breast surgery^12^ and most patients have surgery by 6 months following diagnosis.

### 2.4 Independent variables

We categorized patient age at diagnosis as 18–30, 31–35, 36–40, 41–45 years and registry‐documented marital status as married/partnered, unmarried/widowed/ divorced, and unknown. Race and ethnicity were identified in the cancer registry based on the review of medical record face sheets by hospitals' tumor registrars. Where multiple races were listed in the registry, we utilized the first listed race (of note, MCR instructions required that if an individual had multiple listed races including White race, White be listed last). If the registry identified an individual as being of “Spanish/Hispanic origin” we classified them as Hispanic regardless of their race. Thus, those described as White in this analysis were identified as non‐Hispanic and White in the registry, and those described as Black in our analysis were identified as non‐Hispanic and Black. The registry additionally derived information on Spanish/Hispanic origin from “stated ethnicity, birthplace, personal history and language spoken, and maiden name/surname” as identified in medical records.^13^ We used both APCD enrollment data and the cancer registry payor information to identify whether patients were enrolled in private insurance or Medicaid on the date of their diagnosis. If they were enrolled in both Medicaid and a commercial plan on their diagnosis date (*n* = 270), we classified them as being Medicaid‐insured. We used zip codes from the APCD to assign study participants a median area‐level household income.^14^

We additionally identified whether a patient's cancer physician was affiliated with an academic institution. For patients who had surgery, we initially identified the surgeon on the first claim with a procedure code of lumpectomy or mastectomy occurring on or after the diagnosis date and documented that surgeon's primary hospital affiliation using publicly available Massachusetts Board of Registration information (*n* = 2083). When we could not identify a surgeon in the claims (*n* = 214), we utilized information on the hospital where the patient underwent surgery. Finally, when a patient did not have a surgical claim or we were unable to identify a surgeon, we identified physicians or hospitals based on medical oncology, chemotherapy, or radiotherapy claims (*n* = 127). We used publicly available classifications of Massachusetts health facilities^15^ to classify hospitals as academic medical centers, other teaching hospitals, or community hospitals.

Breast cancer‐related independent variables from the MCR were cancer stage from 0 to 4 and hormone receptor status. We also included a variable indicating the year of our study period in which the cancer diagnoses occurred.

### 2.5 Analysis

We used descriptive statistics to examine population characteristics and rates of *BRCA1/2* testing. We used bivariate analyses to compare *BRCA1/2* testing rates according to age, race/ethnicity, insurance type, area‐level income distribution, cancer stage, hormone receptor status, and academic affiliation of the patient's surgeon, other cancer physician, or facility. We described semiannual trends in *BRCA1/2* testing within 6 months after diagnosis overall and among age, race/ ethnicity, and insurance groups. We then used multivariable logistic regression to examine sociodemographic and clinical factors associated with receipt of *BRCA1/2* testing within 6 months of diagnosis. We included all examined independent variables in our model. Fewer than 11 patients had missing data for race/ ethnicity, marital status, stage, and area‐level income, respectively; to avoid reporting cells with low numbers, missing patients were grouped with other categories in descriptive analyses and the model as in Table 1. For patients with missing area‐level income data, we used the median income as those patients' income for the continuous income variable in our model and included a variable to indicate missingness in our model. We performed a sensitivity analysis in which we included in the model patients with an unknown affiliation of their cancer physician. Analyses were conducted using SAS version 9.4. Two‐sided *p*‐values <0.05 were considered statistically significant. This study was determined to be exempt by the Harvard Medical School Institutional Review Board.

**TABLE 1:** Women ≤ 45 years old diagnosed with breast cancer in Massachusetts from 2010–2013 and rates of BRCA1/2 testing in the 6 months following diagnosis

Characteristic	Overall	BRCA1/2 Testing in 6 months following diagnosis	
	*N* (% of cohort)	*N* (% testing within group)	*p*‐value^a^
Overall	2424 (100.0)	1331 (54.9)	
Age group			<0.001
18–30	103 (4.2)	64 (62.1)	
31–35	230 (9.5)	147 (63.9)	
36–40	642 (26.5)	414 (64.5)	
41–45	1449 (59.8)	706 (48.7)	
Race/Ethnicity			<0.001
Non‐Hispanic White	1947 (80.3)	1122 (57.6)	
Non‐Hispanic Black	155 (6.4)	55 (35.5)	
Hispanic	152 (6.3)	75 (49.3)	
Other^b^/Unknown^c^	170 (7.0)	79 (46.5)	
Marital status			0.05
Married or Unmarried Partner	1571 (64.8)	891 (56.7)	
Single, including separated/ divorced /widowed	797 (32.9)	410 (51.4)	
Unknown/Missing^c^	56 (2.3)	30 (53.6)	
Insurance type			<0.001
Medicaid	573 (23.6)	232 (40.5)	
Private	1851 (76.4)	1099 (59.4)	
Median area‐level household income quartile^c^			<0.001
1 (≤55,698)	598 (24.7)	270 (45.2)	
2 (55,698, 73,567]	600 (24.8)	329 (54.8)	
3 (73,567, 89,833]	610 (25.2)	355 (58.2)	
4 (>89,833)	616 (25.4)	377 (61.2)	
Cancer stage			<0.001
0	549 (22.6)	234 (42.6)	
1	878 (36.2)	512 (58.3)	
2	704 (29.0)	424 (60.2)	
3	223 (9.2)	131 (58.7)	
4 or Unknown^c^	70 (2.9)	30 (42.9)	
Hormone receptor status			0.90
ER or PR positive	1961 (80.9)	1078 (55.0)	
ER and PR negative/Other/Unknown/Not Done/Missing^d^	463 (19.1)	253 (54.6)	
Provider affiliation			<0.001
Academic Medical Center	1551 (64.0)	900 (58.0)	
Teaching Hospital	335 (13.8)	161 (48.1)	
Community Hospital	538 (22.2)	270 (50.2)	
Year at diagnosis			0.006
2010	639 (26.4)	337 (52.7)	
2011	576 (23.8)	320 (55.6)	
2012	591 (24.4)	301 (50.9)	
2013	618 (25.5)	373 (60.4)	

Abbreviations: ER, estrogen receptor; PR, progesterone receptor.

^a^ *p* for the year of diagnosis based on the (two‐sided) Cochran‐Armitage test of trend; all others are chi‐square tests.

^b^ “Other” race/ ethnicity includes individuals identified in the Massachusetts Cancer Registry as Asian/Pacific Islander, Native American/Alaska Native, and Other race.

^c^ <11 individuals had missing data on race/ ethnicity, marital status, cancer stage, or income. For the individuals with missing median area‐level income, the median of the study population's area‐level income was used.

^d^ 28 individuals had hormone receptor status that was classified unknown/ not done/ missing or other.

## 3 RESULTS

Our study population included 2424 women diagnosed with breast cancer between 2010 and 2013 (Figure 1). Table 1 shows patient characteristics and unadjusted associations of patient and tumor characteristics with *BRCA1/2* testing. Almost 60% of women were 41–45 years old, and 26.5% were 36–40 years old. Over 80% of patients were non‐Hispanic White, with 6.4% non‐Hispanic Black and 6.3% Hispanic. Three‐quarters of patients were privately insured, and one‐quarter were insured by Medicaid at the time of diagnosis. Overall, 54.9% of the cohort had *BRCA1/2* testing within 6 months of breast cancer diagnosis.

In unadjusted analyses, *BRCA1/2* testing varied significantly across age groups (*p* < 0.001), occurring less frequently among women aged 41–45 (48.7%) compared to women in the younger age groups (62.1%–65.9%; *p* < 0.001). Married/partnered women had higher rates of testing than unmarried women, although this difference was of borderline statistical significance (*p* = 0.05). *BRCA1/2* testing varied by race/ethnicity (*p* < 0.001) and was less frequent among non‐Hispanic Black women (35.5%) and Hispanic women (49.3%) compared with non‐Hispanic White women (57.6%). Women insured by Medicaid were less likely to receive testing (40.5%) than privately‐insured women (59.4%; *p* < 0.001). Testing also varied according to area‐level income quartile (*p* < 0.001), occurring in 45.2% of those living in the lowest quartile of area‐level income but 61.2% of those in the highest area‐level income quartile. Testing was also associated with cancer stage (*p* < 0.001), with lower testing among women with stage 0 (42.6%) or stage 4/ unknown stage disease (42.9%) compared with Stage 1–3 disease (58.3%–60.2%). Testing rates were higher among women treated by a surgeon or other oncology physician affiliated with an academic medical center (58.0%) compared to those with surgeons affiliated with other teaching hospitals (48.1%) or community hospitals (50.2%; *p* < 0.001). Rates of testing varied by year of diagnosis (*p* = 0.006) and were highest in 2013.

Table 2 shows adjusted associations of patient characteristics with *BRCA1/2* testing. When adjusting for other factors, women aged ≤40 had about twice the odds of being tested than women aged 41–45. Marital status was not statistically significantly associated with testing in adjusted analyses. Compared with non‐Hispanic White women, non‐Hispanic Black women remained less likely to receive testing (adjusted odds ratio [OR] 0.45, 95% CI [0.31, 0.64]) as were women of “other” or unknown race/ethnicity (OR 0.58, 95% CI [0.42, 0.81]). Hispanic women had similar adjusted odds of testing compared with non‐Hispanic White women. Women insured by Medicaid had about half the adjusted odds of testing as privately‐insured women (OR 0.51, 95% CI [0.41, 0.63]). Residing in higher‐income versus lower‐income areas was associated with a greater likelihood of testing (OR 1.57 [95% CI 1.21, 2.04] for the highest versus lowest quartile). Having stage 4 or unknown stage cancer was associated with a lower likelihood of testing compared with stage 0 disease (OR 0.50 95% CI [0.29, 0.83]). Being treated by a physician affiliated with an academic medical center was not associated with receipt of testing when adjusting for other variables. Testing remained more likely for patients diagnosed in 2013 compared to 2010 (OR 1.45 [95% CI 1.14, 1.84]). In sensitivity analyses including the 166 patients for whom we were not able to identify whether their provider or facility was academically affiliated, results were very similar. (Appendix S2).

**TABLE 2:** Multivariable logistic regression model examining adjusted associations of patient and provider characteristics with *BRCA1/2* testing within 6 months of breast cancer diagnosis

Characteristic	OR (95% CI)
Age	
18–30	**1.99 (1.28, 3.10)**
31–35	**2.00 (1.47, 2.71)**
36–40	**2.05 (1.67, 2.51)**
41–45	Ref
Race/Ethnicity	
Non‐Hispanic White	Ref
Non‐Hispanic Black	**0.45 (0.31, 0.64)**
Hispanic	0.94 (0.65, 1.36)
Other/Unknown	**0.58 (0.42, 0.81)**
Marital status	
Married or Partnered	Ref
Single/Separated/Divorced/ Widowed	1.05 (0.86, 1.28)
Unknown/Missing	1.04 (0.59, 1.85)
Insurance type	
Medicaid	**0.51 (0.41, 0.64)**
Private	Ref
Median area‐level household income– Quartiles^a^	
1 (≤55,698)	Ref
2 (55,698, 73,567]	1.24 (0.97, 1.59)
3 (73,567, 89,833]	**1.31 (1.02, 1.69)**
4 (>89,833)	**1.57 (1.21, 2.04)**
Cancer stage	
0	**0.53 (0.42, 0.66)**
1	Ref
2	1.06 (0.85, 1.31)
3	1.05 (0.76, 1.44)
4 or Unknown	**0.50 (0.29, 0.84)**
Hormone receptor status	
ER or PR positive	Ref
ER and PR negative/Other/Unknown/Not Done/Missing	0.90 (0.72, 1.12)
Physician hospital affiliation	
Academic Medical Center	1.06 (0.86, 1.32)
Teaching Hospital	0.95 (0.71, 1.26)
Community Hospital	Ref
Year at diagnosis	
2010	Ref
2011	1.16 (0.92, 1.47)
2012	0.93 (0.74, 1.18)
2013	**1.45 (1.14, 1.84)**

Abbreviations: ER, estrogen receptor; PR, progesterone receptor.

^a^ The model also included a variable for “Unknown area‐level income” (versus known). The OR (95% CI) was 3.80 (0.35, 40.94). Bold values indicate statistical significance at the 0.05 level.

## 4 DISCUSSION

In this population‐based study of Massachusetts women aged 45 years and under with breast cancer, we found that non‐Hispanic Black women, Medicaid‐insured women, and women living in lower‐income areas were statistically less likely than White, privately insured women, and women living in higher‐income areas, respectively, to receive guideline‐concordant *BRCA1/2* testing within 6 months of diagnosis, adjusting for other factors. Testing among women aged 41–45 was less frequent than among younger women, perhaps due to a lag in the uptake of the NCCN's expansion of age criteria in 2009.

Our findings are consistent with other studies demonstrating racial/ ethnic and socioeconomic disparities in *BRCA1/2* testing among individuals with cancer. One study using administrative claims data from a national sample of commercially‐insured women ≤40 years old with breast cancer during 2004–2007 found that racial/ethnic minority women had lower rates of testing than White women.^16^ A study using surveys combined with registry data of more than 3000 women in Pennsylvania and Florida from 2007–2009 demonstrated that Black women were less frequently recommended to have *BRCA1/2* testing than White women, regardless of their risk for having a mutation.^4^ A large population‐based study from California and Georgia in 2013–2014 found no racial/ethnic differences in genetic testing rates among women with breast cancer across all ages, but among women aged ≤45 years, 68% of Black versus 78% of White women had testing.^17^ A number of hypotheses for racial/ethnic inequities have been proposed, including limited clinician and patient awareness about *BRCA1/2* risk among racially and ethnically diverse populations, clinician bias, and patient trust. Our findings that insurance and area‐level income were strongly associated with testing suggest that socioeconomic barriers to high‐quality services persisted in Massachusetts during this study period, despite insurance coverage and Massachusetts' generally positive record on health care access. Clinicians' perceptions of coverage for and affordability of testing may have also played a role in lower testing rates among lower‐income and Medicaid‐insured women. Evidence from other states suggests that lower‐income, Medicaid‐insured, and Black women may have also received care from different providers than higher‐income, privately‐insured, and White women,^4^ although clinicians' academic affiliation was not associated with the likelihood of testing when adjusting for patient characteristics in this study.

Our study has several strengths. Our use of the Massachusetts Cancer Registry allowed us to identify all cases of breast cancer diagnosed in young women in Massachusetts in our study period, while linkage with the APCD allowed us to capture all health care utilization that was paid for by both Medicaid and private insurance. To our knowledge, linkage of a state cancer registry and its APCD has been described in the literature in only one other state, Utah,^18^, ^19^ and we are not aware of any prior use of this linkage to assess genetic testing utilization. While the APCD‐registry linkage is operationally complex and time‐consuming, our study demonstrates the promise of such linkages in examining health care utilization among a large, diverse population with cancer.

However, our study also has several limitations. First, the use of claims data does not permit examination of testing not covered by insurance. If patients paid for testing out‐of‐pocket or received free testing through genetic testing companies' charity programs, such testing would not be evident in claims. However, it is very likely that *BRCA1/2* testing among those with breast cancer was covered during this time period by private insurers, and personal communication with Massachusetts Medicaid officials confirmed that it was covered by Massachusetts Medicaid. Second, findings from Massachusetts may not generalize to other states, including states with less generous insurance coverage or more rural areas, where access to genetic testing may be less robust and disparities may be even more pronounced. Our study also does not address the experience of women insured by Medicare (for example due to disability) or who are uninsured, although <4% of individuals in Massachusetts were uninsured during this time period.^20^ Third, women in our cohort were diagnosed with cancer in 2010–2013, prior to important changes in *BRCA* test access.^21^ This time period had the advantage of allowing for complete claims capture for privately insured women because it preceded the Gobeille vs. Liberty Mutual Supreme Court decision that allowed self‐insured plans to opt out of submitting claims to state APCDs.^22^ However, whether testing rates have increased and whether disparities have narrowed or widened in Massachusetts with increased access to testing is not known; updated analyses will be critically important to examine these trends. Finally, claims data typically provide limited information on the health care providers caring for patients at their time of diagnosis. Since clinician factors, including the availability of genetic counselors, may play a key role in exacerbating or mitigating testing inequities, further research should gather more robust provider‐level data.

In summary, this study using a distinctive linkage of APCD data and state cancer registry data demonstrates that even in a state with relatively generous insurance coverage and access to high‐quality cancer care, concerning racial/ethnic and socioeconomic disparities exist in guideline‐concordant genetic testing among young women with breast cancer. Racial/ethnic disparities in breast cancer outcomes persist in Massachusetts, with Black women at a higher likelihood of dying from breast cancer than White women in 2013–2017.^23^ Addressing disparities in the receipt of high‐quality cancer care, including indicated genetic testing, may be an important component of strategies to reduce racial, ethnic and socioeconomic inequities in outcomes.

## CONFLICT OF INTEREST

The authors declare that they have no conflicts of interest.

## AUTHOR CONTRIBUTIONS

Conception and design: LEP, NLK, SSH. Data assembly: NLK, RW, RK, SG, JZA, JW. Data analysis and interpretation: all authors. Manuscript writing: LEP, NLK. Manuscript review: all authors.

## ETHICS STATEMENT

This study was determined to be exempt by the Harvard Medical School Institutional Review Board.

## Supporting information


Appendix S1
Appendix S2

## Data Availability

The data that support the findings of this study were provided to the authors by the Massachusetts Center for Health Information and Analysis and the Massachusetts Cancer Registry. The data are not available from the authors due to restrictions in the Data Use Agreement.
