# Supplementary material for: BRCA1/2 testing among young women with breast cancer in Massachusetts, 2010–2013: An observational study using state cancer registry and All‐Payer claims data
Source: Cancer Med. 2022 Mar 21;11(13):2679–86. doi: 10.1002/cam4.4648 (PMC9249986; doi:10.1002/cam4.4648)
Supplement: Supplementary file 1 — Appendix S1 Appendix S2 [file CAM4-11-2679-s001.docx]

**Supplemental Materials**

**Appendix A. Linkage of Massachusetts Cancer Registry (MCR) Data with Massachusetts All Payer Claims Data (APCD)**

We identified all 83,105 breast, colorectal, lung, or prostate cancer cases diagnosed from 2010-2013 among individuals aged ≥18 years in the MCR data. We obtained annual member eligibility files for 2010-2014 from the APCD from the Massachusetts Center for Health Information and Analysis (CHIA) eligibility files. We used Link Plus (https://www.cdc.gov/cancer/npcr/tools/registryplus/lp.htm), a probabilistic record linkage program developed by the Centers for Disease Control, to match registry cases to the APCD. Link Plus sets weights for matching values based on the frequencies of values in the files being compared. A match on a frequent value is associated with a low weight, whereas a match on a rare value is associated with a high weight. Partial matching on separate date components, transposition of date components, and missing month or day values are taken into account as are differences in Social Security numbers and Zip codes.

Each year’s APCD member eligibility file included approximately 8.5 million records with a unique member eligibility identifier (MEID); combining these data across 2010-2014, we identified approximately 11 million unique records. This final member eligibility file was then matched with the MCR cancer case file using Link Plus and used 7 matching variables: last name, first name, middle initial, date of birth, Social Security number, sex, and Zip code.

Link Plus was run with its recommended cutoff score of 10. Most of the matches with scores between 10 and 18.9 matched on only one element of the name or date of birth and not on Social Security number. To finalize decisions about the best threshold score, we visually reviewed matches with scores between 14.0 and 22.0 and found a striking drop off in the likelihood of an actual match below 18.9 (eTable 1). Using a threshold score of 18.9 or higher, 94% to 100% of the matches appeared to be true matches.

eTable 1. Summary of Visual Review of Matches with Threshold Score Between 14-22

| **Threshold Score** | **N LinkPlus Matches** | **N Matches on Visual Review** | **% Agreement - Visual/LinkPlus** |
| --- | --- | --- | --- |
| 22.0 | 144 | 144 | 100% |
| 19.9 | 107 | 107 | 100% |
| 19.8 | 76 | 75 | 99% |
| 19.7 | 67 | 64 | 96% |
| 19.6 | 58 | 58 | 100% |
| 19.5 | 64 | 63 | 98% |
| 19.4 | 137 | 133 | 97% |
| 19.3 | 115 | 112 | 97% |
| 19.2 | 70 | 66 | 94% |
| 19.1 | 171 | 167 | 98% |
| 19.0 | 69 | 68 | 99% |
| 18.9 | 78 | 76 | 97% |
| 18.8 | 116 | 43 | 37% |
| 17.9 | 67 | 20 | 30% |
| 16.9 | 91 | 20 | 22% |
| 16.0 | 115 | 13 | 11% |
| 14.9 | 142 | 3 | 2% |
| 14.8 | 124 | 6 | 5% |
| 14.7 | 142 | 5 | 4% |
| 14.6 | 121 | 9 | 7% |
| 14.5 | 162 | 9 | 6% |
| 14.4 | 174 | 2 | 1% |
| 14.3 | 166 | 4 | 2% |
| 14.2 | 193 | 6 | 3% |
| 14.1 | 194 | 0 | 0% |
| 14.0 | 234 | 8 | 3% |

When assigning ≥18.9 as the threshold, 77,299 of 83,105 cancer cases in the registry had at least one match (93.0%). Of note, with lower linkage threshold scores we observed more duplicate matches (patients who matched to >1 MEID) (eTable 2). We believe this was due to duplicates in our updated member eligibility file that combined across years (which included about 11 million unique records), since CHIA staff noted that yearly member eligibility totals (using unique occurrences of member link ID) are approximately 7-8 million per year which was comparable to our yearly medical eligibility totals.

eTable 2. Summary of LinkPlus Matching results

| **Threshold Score** | **Total Matches** | **Cumulative Unique Matches+** | **New Matches** | **Patients Added** | **Duplicate Matches Added** | **N Patients Remaining Unmatched** | **% with ≥1 match** |
| --- | --- | --- | --- | --- | --- | --- | --- |
| 43.1 | 1028 | 1028 | 1028 | 1028 | 0 | 82077 | 1.2% |
| 43 | 1103 | 1103 | 75 | 75 | 0 | 82002 | 1.3% |
| 42 | 2527 | 2527 | 1424 | 1424 | 0 | 80578 | 3.0% |
| 41 | 4975 | 4973 | 2448 | 2446 | 2 | 78132 | 6.0% |
| 40 | 13062 | 13056 | 8087 | 8083 | 4 | 70049 | 15.7% |
| 39 | 21288 | 21279 | 8226 | 8223 | 3 | 61826 | 25.6% |
| 38 | 29659 | 29649 | 8475 | 8474 | 1 | 53352 | 35.7% |
| 37 | 37249 | 37235 | 7591 | 7587 | 4 | 45766 | 44.8% |
| 36 | 43359 | 43338 | 6110 | 6103 | 7 | 39663 | 52.1% |
| 35 | 46694 | 46664 | 3335 | 3326 | 9 | 36337 | 56.1% |
| 34 | 48983 | 48940 | 2289 | 2276 | 13 | 34061 | 58.9% |
| 33 | 50765 | 50703 | 1782 | 1763 | 19 | 32298 | 61.0% |
| 32 | 52895 | 52755 | 2130 | 2052 | 78 | 30246 | 63.5% |
| 31 | 55013 | 54788 | 2118 | 2033 | 85 | 28213 | 65.9% |
| 30 | 58819 | 58313 | 3806 | 3525 | 281 | 24688 | 70.2% |
| 29 | 62688 | 61800 | 3869 | 3487 | 382 | 21201 | 74.4% |
| 28 | 66586 | 65293 | 3898 | 3493 | 405 | 17708 | 78.6% |
| 27 | 69928 | 68156 | 3342 | 2863 | 479 | 14845 | 82.0% |
| 26 | 73652 | 71036 | 3724 | 2879 | 844 | 11966 | 85.5% |
| 25 | 76146 | 72889 | 2494 | 1853 | 641 | 10113 | 87.7% |
| 24 | 77965 | 74176 | 1819 | 1287 | 532 | 8826 | 89.3% |
| 23 | 79375 | 75065 | 1410 | 889 | 521 | 7937 | 90.3% |
| 22 | 80726 | 75876 | 1351 | 811 | 540 | 7126 | 91.3% |
| 21 | 81766 | 76382 | 1040 | 506 | 534 | 6620 | 91.9% |
| 20 | 82570 | 76773 | 805 | 391 | 413 | 6229 | 92.4% |
| 19 | 83494 | 77159 | 955 | 391 | 538 | 5838 | 92.8% |
| **18.9** | **83710** | **77299** | **79** | **31** | **48** | **5807** | **93.0%** |
| 18-18.8 | 84385 | 77496 | 812 | 306 | 506 | 5501 | 93.4% |
| 17 | 85084 | 77706 | 699 | 210 | 489 | 5399 | 93.5% |
| 16 | 85878 | 77851 | 794 | 145 | 649 | 5254 | 93.7% |
| 15 | 87030 | 77969 | 1152 | 118 | 1034 | 5136 | 93.8% |
| 14 | 88711 | 78061 | 1681 | 92 | 1589 | 5044 | 93.9% |

*Threshold scores <14 omitted; for these we observed substantially fewer matches.

+-indicates than one or more MCR cases matched.

Of these 77,299 cancer cases, 5807 matched to more than one MEID (83,710 total matches). There were 5,364 cancer cases that matched to 2 MEIDs, 330 that matched to 3 MEIDs, 52 that matched to 4 MEIDs, 12 that matched to 5 MEIDs, 7 that matched to 6 MEIDs, 5 that matched to 7 MEIDs, and one each that matched to 8 and 9 MEIDs.

In many circumstances one cancer case matched perfectly on all the elements to one MEID and matched on all the variables except SSN (either missing in the medical eligibility file or different in the member eligibility file) to another MEID. Even among matches without the SSN or a different SSN in the medical eligibility file, visual review determined that most were matches due to the uniqueness of the first name and/or surname. We further verified that the vast majority of cases that linked to more than one MEID from the medical eligibility file linked to a single set of medical claims (even if they had 2 or more MEIDs). Thus, we determined to keep all matches with link score ≥18.9. Among the duplicates, we deleted any duplicates for which there were no claims (most duplicates) and those with no claims from 30 days before diagnosis through 180 days after diagnosis or for whom there were claims after the date of death, as these would be evidence of an erroneous match, and we retained the remaining claims.

After determining that the original registry data included 4,587 gastrointestinal cancer cases that were not colorectal cancers, we limited the Massachusetts Cancer Registry file to the 78,518 cases instead of the original 83,105 cases. We identified 73,013 cancer cases that matched to at least one MEID (79,034 total matches) (eFigure 1). These matched APCD records included 1,332,333,288 Medicaid and Commercial medical claims and 723,149,920 Medicaid and Commercial pharmacy claims.

eFigure 1. Summary of Matching Cancer Registry and All Payer Claims Data


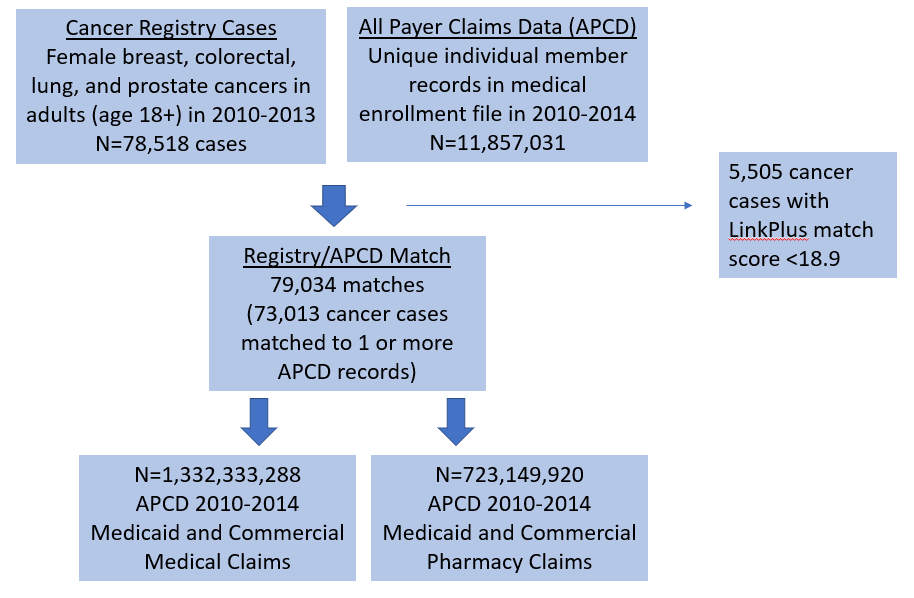


**Appendix B. Multivariable logistic regression model examining adjusted associations of patient and provider characteristics with *BRCA1/2* testing within 6 months of breast cancer diagnosis, including patients for whom hospital affiliation could not be determined (n=166)**

| **Characteristic** | **OR (95% CI)** |
| --- | --- |
| **Age** |  |
| 18-30 | **2.02 (1.30,3.12)** |
| 31-35 | **2.04 (1.52, 2.75)** |
| 36-40 | **1.98 (1.63,2.42)** |
| 41-45 | **Ref** |
| **Year at Diagnosis** |  |
| 2010 | **Ref** |
| 2011 | 1.16 (0.92, 1.46) |
| 2012 | 0.93 (0.74,1.17) |
| 2013 | **1.48 (1.17,1.88)** |
| **Race/ ethnicity** |  |
| Non-Hispanic White | **Ref** |
| Non-Hispanic Black | **0.46 (0.32,0.66)** |
| Hispanic | 1.02 (0.71,1.46) |
| Other / Unknown | **0.57 (0.41,0.79)** |
| **Marital Status** |  |
| Married or Partnered | **Ref** |
| Single/Separated/Divorced/ Widowed | 1.02 (0.84,1.24) |
| Unknown/Missing | 0.96 (0.55,1.68) |
| **Median area-level household income/ 100,000 – Quartiles*** |  |
| 1 (<=55698) | **Ref** |
| 2 (55698,73567] | 1.25 (0.98,1.59) |
| 3 (73567,89833] | **1.30 (1.01,1.67)** |
| 4 ( > 89833) | **1.56 (1.20,2.02)** |
| **Insurance type** |  |
| MassHealth | **0.50 (0.40,0.63)** |
| Private | **Ref** |
| **Physician hospital affiliation** |  |
| Academic Medical Center | 1.06 (0.86, 1.31) |
| Teaching Hospital | 0.94 (0.71,1.26) |
| Community Hospital | **Ref** |
| Unknown affiliation | **0.11 (0.07,0.18)** |
| **Cancer Stage** |  |
| 0 | **0.53 (0.43,0.66)** |
| 1 | **Ref** |
| 2 | 1.03 (0.84,1.28) |
| 3 | 1.05 (0.77,1.44) |
| 4 or Unknown | **0.56 (0.34,0.93)** |
| **Hormone Receptor Status** |  |
| ER or PR positive | **Ref** |
| ER and PR negative / Other / Unknown / Not Done / Missing | 0.91 (0.73,1.13) |

ER = estrogen receptor; PR= progesterone receptor

*The model also included a variable for “Unknown area-level income” (versus known). The OR (95% CI) was 3.70 (0.35, 39.62).
